# Accumulation of CO_2_ limits energy gain in freely diving grey seals

**DOI:** 10.1242/jeb.251718

**Published:** 2026-05-08

**Authors:** Eva-Maria S. Bønnelycke, Joanna L. Kershaw, Gordon D. Hastie, Carol Sparling, Steve Balfour, Ryan Milne, Simon E. W. Moss, Philippa F. C. Wright, J. Chris McKnight

**Affiliations:** ^1^ Sea Mammal Research Unit, Scottish Oceans Institute, University of St Andrews, St Andrews, Fife KY16 8LB, UK; ^2^ Sea Mammal Research Unit Instrumentation Group, Scottish Oceans Institute, University of St Andrews, St Andrews, Fife KY16 8LB, UK

**Keywords:** Energetics, Phocid seals, Respirometry, Aerobic dive limit, Lactate, Digestion

## Abstract

Understanding how gas regulation impacts behavioural and physiological processes in phocid seals is essential to understanding their foraging ecology. The accumulation of circulating CO_2_ across a series of dives is thought to prolong surface recovery, thereby reducing foraging efficiency. This can be empirically tested by experimentally altering circulating gas tensions in diving seals and quantifying the effect on net rate of energy gain. In the present study, six grey seals (*Halichoerus grypus*) voluntarily dove in a simulated foraging setup, swimming from a breathing chamber to and from an underwater feeder. During surface intervals, seals were exposed to ambient, hypercapnic (high CO_2_), hypoxic (low O_2_) or hyperoxic (high O_2_) respiratory gas conditions. The effect of gas condition on net rate of energy gain, dive behaviour, respirometry-derived energy expenditure, post-dive circulating lactate concentration, and digestion (indicated by circulating triglyceride concentrations) was quantified. Net rate of energy gain significantly decreased under hypercapnia, likely owing to extended surface recovery durations, rather than underlying changes in energy expenditure or other post-dive metabolic processes. Extended surface durations reflected the slower rate of CO_2_ elimination relative to O_2_ uptake. Our findings show that the accumulation of CO_2_ is a significant limiting factor to net rate of energy gain in grey seals. Furthermore, we provide evidence of both digestion and anaerobic metabolism during diving, which contrasts with previously hypothesised optimal foraging strategies. Phocid seals are therefore not limited by digestive activity or the accumulation of lactate during short foraging bouts.

## Teaser

**Summary:** Optimal foraging in phocid seals is influenced by circulating gas tensions. The accumulation of CO_2_ affecting surface interval duration limits foraging efficiency in grey seals.

## INTRODUCTION

Phocid seals have unique anatomical and physiological adaptations to repeatedly dive to depth and periodically surface to breathe. For example, southern elephant seals (*Mirounga leonina*) spend 78% of their lives underwater, routinely diving to depths of 300–700 m for 20–30 min while requiring less than 4 min to recover at the surface before undertaking subsequent dives (Hindell et al., 1992; McIntyre et al., 2010; Slip et al., 1994). Breath-hold diving causes a decline in circulating O_2_ (hypoxia) and an elevation in circulating CO_2_ (hypercapnia), which are normal physiological shifts during routine diving of phocid seals. In theory, gas exchange at the surface restores O_2_ and eliminates accumulated CO_2_ upon resumption of breathing after individual dives. However, previous work in grey seals (*Halichoerus grypus*), Weddell seals (*Leptonychotes weddellii*) and Steller sea lions (*Eumetopias jubatus*) has shown that these animals often incur an O_2_ debt and accumulation of CO_2_ over the course of a series of dives in a diving bout with relatively short inter-dive surface intervals (Boutilier et al., 2001; Fahlman et al., 2008; Falke et al., 2008; Gerlinsky et al., 2014; Kooyman et al., 1973; Rosen et al., 2017). During these diving bouts, gas exchange is completed in a longer post-dive surface recovery period following the last dive. Although the decline in circulating O_2_ limits dive duration, the accumulation of CO_2_ is thought to have a greater influence on the surface interval duration (Gerlinsky et al., 2014; Kooyman and Ponganis, 1998). After surfacing with an O_2_ debt, the leftward shift in the oxygen-dissociation curve means haemoglobin binds O_2_ more readily at lower partial pressures of O_2_. As such, haemoglobin's increased affinity for O_2_, and the resulting alveolar-to-blood diffusion gradient, facilitates rapid O_2_ uptake. CO_2_, however, is primarily stored as bicarbonate and H^+^, and the biochemical conversion back to CO_2_ slows its removal from the body in exhaled breath. Overall, this results in a slower rate of CO_2_ elimination relative to O_2_ uptake at the surface.

The marked changes in circulating blood gas tensions experienced by diving seals can be experimentally induced by modifying the percentage of O_2_ and CO_2_ through inhaled respiratory gas exposures. This method has been used to study the hypercapnic and hypoxic respiratory, heart rate and behavioural responses in several diving vertebrate species (e.g. Borg et al., 2004; Glass and Johansen, 1979; Stone et al., 1992), including marine mammals (e.g. Bainton et al., 1973; Gallivan, 1980; Gerlinsky et al., 2014; Parkos and Wahrenbrock, 1987; Påsche, 1976). Understanding how changes in circulating O_2_ and CO_2_ levels impact behavioural and physiological processes in marine mammals is essential to understanding how they maximise prey acquisition during foraging, and how they may respond to or compensate for any disruptions to foraging. However, the trade-offs between gas management and other physiological demands in shaping behavioural decisions remains overlooked in their biology.

Optimal foraging theory predicts the behaviours animals employ to maximise their net rate of energy gain (hereafter termed ‘energy gain’); for seals, the physiological constraints imposed by circulating O_2_ and CO_2_ tensions on their diving behaviour (i.e. dive and surface recovery duration) fundamentally limit the time available for foraging such that it is likely to affect overall foraging efficiency (Foo et al., 2016). During foraging trips, seals must adapt their diving behaviour under dynamic situations, for example by extending dives if a dense prey patch is encountered (Sparling et al., 2007a). By investigating the influence of circulating gas tensions on dive behaviour, and in turn metabolic rate and energy gain, we can gain a better understanding of the drivers of short-term behavioural decision-making influencing how seals maximise prey yield, behavioural efficiency and metabolic efficiency during foraging. Indeed, previous work suggests that the accumulation of CO_2_ during diving bouts limits foraging efficiency because more time is needed to offload CO_2_ than to load O_2_ at the surface, thereby reducing the amount of time spent underwater (Gerlinsky et al., 2014). This can be empirically tested by quantifying the effect of experimentally altered circulating gas levels and subsequent behavioural responses on energy gain, which serves as a functional measure of foraging efficiency by accounting for the energetic expenditure during diving and energy intake from prey acquisition. However, to our knowledge, the effect of hypercapnia on energy gain has not yet been quantified.

To understand the effect of circulating gas tensions on energy gain, post-dive metabolic processes such as the accumulation and clearance of metabolic byproducts, including lactate, and the timing of digestion should also be considered as they contribute to overall energy expenditure and potentially influence dive behaviour. Simulations and empirical tests of diving optimality models suggest that phocid seals maximise energy gain by completing the majority of their dives within the aerobic dive limit, such that they do not deplete their oxygen stores and avoid the subsequent reliance on anaerobic metabolism, which causes lactate accumulation and extended recovery times (Sparling et al., 2007a; Thompson and Fedak, 2001). Similarly, the timing of digestion may reflect a strategy to minimise energetic costs during diving to maximise foraging time. For example, previous work in grey seals suggests that they may defer the timing of digestion up to several hours after a feeding bout to preserve the associated metabolic requirements and oxygen demands needed to optimise dive performance during foraging (Sparling et al., 2007b). Although much of the research on foraging behaviour has focused on O_2_ as a primary limiting factor, the extent to which CO_2_ accumulation influences dive decisions, and the relative energetic costs of post-dive recovery from CO_2_ clearance, anaerobic metabolism and digestion, remain poorly understood.

The aim of the present study was to assess how changes in physiological state induced by varying respiratory gas conditions, and thereby circulating gas tensions, affect energy gain of freely diving grey seals. Voluntarily diving grey seals were exposed to pre-determined respiratory gas conditions in a simulated foraging setup where the number of fish consumed and their energy density was recorded. The behavioural responses to the specified gas mixes used in this study were previously reported in McKnight et al. (2025) as evidence for cognitive perception of circulating O_2_ in seals; here, we build on those findings to provide context for how elevated circulating CO_2_ may influence energy gain. In the present study, we assessed changes in the clearance of metabolic byproducts and the timing of digestion by modelling responses of the following physiological metrics to different gas conditions: (1) respirometry-derived metabolic rates to estimate energy expenditure across entire diving bouts, (2) measures of lactate concentration at baseline and immediately post-diving to infer whether gas conditions caused a metabolic shift towards anaerobic metabolism during diving, and (3) measures of circulating triglyceride concentration in serial blood samples to investigate whether gas condition affected the timing of digestion of fish consumed during the trial. The consequences of these responses were then linked to changes in energy gain by combining measures of energy expenditure and the number and energy density of fish consumed per diving trial.

**Table:** 

**List of symbols and abbreviations**	
ADL	aerobic dive limit
cADL	calculated aerobic dive limit
IDSI	inter-dive surface interval
RER	respiratory exchange ratio
RQ	respiratory quotient
TAG	triglyceride concentration immediately post-diving
*V*_CO_2__	volume of carbon dioxide produced
*V*_O_2__	volume of oxygen consumed
*V̇*_CO_2__	mass-specific rate of carbon dioxide production
*V̇*_O_2__	mass-specific rate of oxygen consumption
ΔTAG	change in triglyceride concentration between immediately post-diving and 3 h post-diving

## MATERIALS AND METHODS

### Ethics statement

All procedures for capture, handling and housing of animals were carried out under UK Home Office project licence PP0562940 under the Animals (Scientific Procedures) Act 1986.

### Animals

Six wild-captured grey seals [*Halichoerus grypus* (Fabricius 1791); three males and three females aged 5–7 months] were temporarily housed at the Sea Mammal Research Unit Pool Facility for a period of 13 months. The seals had access to outdoor seawater pools maintained at ambient seawater temperature ranging from 6 to 12°C over the course of the experiments. Seals were fed a daily diet of herring (*Clupea harengus*) and sprat (*Sprattus sprattus*) supplemented with vitamins (Zoovet Aquavits and Ferrous Gluconate, International Zoo Veterinary Group, Keighley, UK) and were trained to complete husbandry protocols and move between areas of the pool facility using positive reinforcement. Throughout their time at the facility, animals were fed individualised amounts of fish based on their body mass to maintain a rate of mass gain consistent with that observed in wild juvenile seals. However, when individuals were involved in experimental trials for 1–2 weeks at a time, they would each receive a daily ration of 2 kg of fish per day (as explained below). Upon completion of the experiments, seals were health checked and released back into the wild at the site of capture.

### Experimental protocol (simulated foraging setup)

**Fig. 1.**
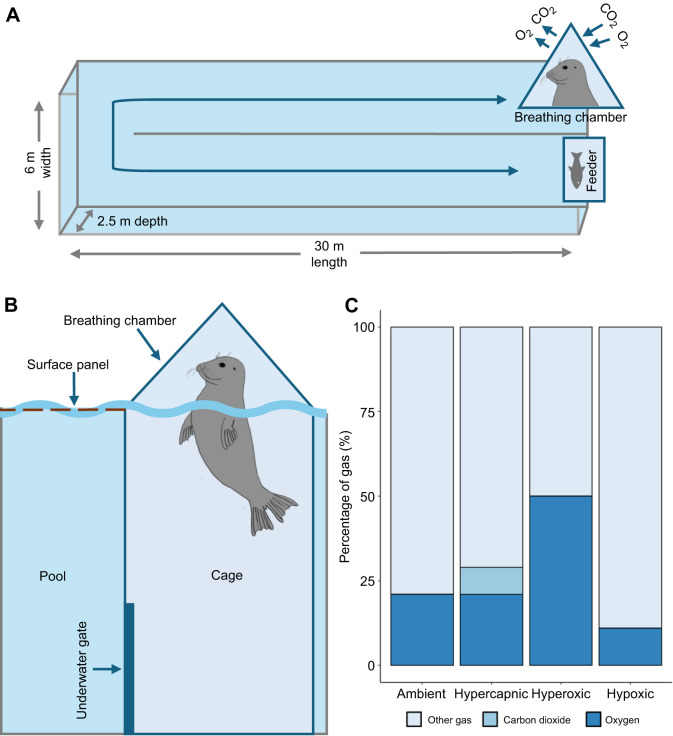
**Visualisation of the simulated foraging setup and respiratory gas conditions.** (A) Schematic plan view of the experimental setup in which a seal repeatedly transits underwater to a feeder and returns to the surface in a breathing chamber. (B) Diagram of the breathing chamber and attached cage with the underwater gate. (C) The percentage of gas (%) for each respiratory gas condition delivered in the breathing chamber during each of the four different experimental treatments.

For the experimental protocol, seals were familiarised with a simulated underwater foraging setup. Animals would complete a 60 m underwater transit from a breathing chamber at the surface to an underwater feeding station and back (Fig. 1A). This transit distance is consistent with dive depths recorded for free-ranging seals in the UK (McConnell et al., 1999; Thompson and Fedak, 1993; Thompson et al., 1991), albeit that in the present study, animals completed horizontal dives rather than vertical dives. Only one seal would partake in the simulated foraging setup at a time. The experimental pool (30×6×2.5 m) was covered with sub-surface aluminium mesh panels, thereby restricting surfacing to the breathing chamber. The underwater feeding station consisted of a conveyer belt that presented sprat to the seals at a set delivery rate to simulate a constant density prey patch. The breathing chamber consisted of a clear acrylic pyramid fixed to an aluminium cage with an underwater gate (Fig. 1B). Additionally, the breathing chamber was connected to an open-flow respirometry system (see ‘Open-flow respirometry’). For more details on the simulated foraging setup, see Sparling and Fedak (2004) and Sparling et al. (2007a).

A series of consecutive dives on a single day in the simulated foraging setup is referred to as a diving trial. At the start of each diving trial, a single seal entered the experimental pool. Access was restricted to the breathing chamber and attached cage for an ∼5 min acclimation period (Fig. 1B). After this, the gate was opened and the seal was given access to the whole pool, where it was free to make repeated dives between the feeder and the breathing chamber at its own volition. The seals were free to determine how long they remained at the feeder, how fast they transited to and from the feeder, and how long they remained at the surface breathing. Although 1.5 kg of sprat was available for each diving trial, trials were terminated before the full 1.5 kg of sprat was consumed to avoid animals finishing their ration of sprat before the end of their last dive. As such, the experimental trial was complete once the seals were close to finishing their daily ration of sprat from the underwater feeder, meaning the number of fish consumed differed for each diving trial. The remainder of the daily sprat ration was then offered to the individual seals after the diving trial. The 1.5 kg of sprat consumed during diving trials was not representative of the total daily ration of fish, and therefore the seals were likely not reaching satiation and maintained motivation during diving trials. On completion of their final dive in a trial, seals were restricted to the breathing chamber and attached cage. Seals were either left for an ∼5 min recovery period or they were immediately lifted out of the pool to obtain a post-diving mixed venous blood sample. These two scenarios were used to prioritise either obtaining post-diving blood samples, which required seals to be lifted to the surface, or to collect extended respirometry measurements during recovery, which required seals to remain in the breathing chamber.

Half of the diving trials included the extended post-diving recovery period in the breathing chamber to account for the post-diving O_2_ debt. The ∼5 min duration of the recovery period was chosen to balance maximising the recovery time in the breathing chamber with limiting the amount of time that seals were confined to the breathing chamber and attached cage. As such, the overall metabolic cost of the entire diving bout may not have been captured in all diving trials with the extended post-diving recovery period. The other half of the diving trials included the post-diving blood sampling within 2 min of the seals' return from their last dive. These blood samples were used to quantify lactate concentration as an indicator of anaerobic metabolism, and triglyceride concentration as an indicator of digestive activity during diving. After the end of a diving trial, the breathing chamber and attached cage with the seal in it was lifted out of the pool by a crane attached to the cage, and once blood sampling was complete, they moved back into non-experimental pools and haul-out spaces.

Each seal completed diving trials for 1–2 experimental weeks at a time followed by a 2 week ‘rest period’. During experimental weeks, seals were exposed to one of the four respiratory gas conditions each day for 4 days, meaning they completed diving trials in each of the gas conditions every experimental week. On experimental days, the daily food ration was maintained at 1.5 kg sprat and 0.5 kg herring across all seals. The 0.5 kg of herring was given to the seals as part of positive reinforcement for their movement to and from the experimental pool for the simulated foraging setup. When possible, the seals' masses were taken as they crossed static scales before the start of each diving trial to keep a record of changes in mass throughout the experimental period.

### Gas conditions

During surface intervals of diving trials, seals were exposed to one of four respiratory gas conditions: (1) ambient air (21% O_2_ | 0.04% CO_2_), (2) hypercapnic air (21% O_2_ | 8% CO_2_), (3) hypoxic air (11% O_2_ | 0.04% CO_2_) and (4) hyperoxic air (50% O_2_ | 0.04% CO_2_) (Fig. 1C). To assess the impacts of ecologically relevant gas tensions and remain within safe margins, the percentages of inspired O_2_ for the hypoxic condition and CO_2_ for the hypercapnic condition were based on physiological thresholds of circulating and end-tidal gas concentrations previously reported in diving phocid seals (Falke et al., 2008; Meir et al., 2009; Qvist et al., 1986). During the experimental period, exposure to gas condition was randomised across diving trials of the six seals, and gas condition was constant within each diving trial. The gas conditions were controlled through the administration of O_2_, CO_2_, nitrogen and ambient air at pre-determined flow rates to the breathing chamber. Gas flow rates were monitored using flowmeters built into a custom gas control unit designed for the purpose of these experiments (JFD, Aberdeen, UK; McKnight et al., 2025).

Prior to the experiments, seals were exposed to a period of gradual change in gas levels in the breathing chamber to ensure changes in gas concentrations did not induce visible signs of distress. Seals were initially exposed to low levels of gas manipulation for short periods and then conditions were controlled to become increasingly hypercapnic, hypoxic and hyperoxic over the course of 2 months.

### Dive behaviour measurements

During diving trials, footage from underwater cameras (Sony IR 37CSHR-IR 25 m) connected to a DVR was monitored in real time to ensure the seals' safety and record diving behaviour. The start and end times of each dive, inter-dive surface interval (IDSI), and the times at which the seals arrived and left the feeder were recorded. Additionally, the number of individual sprat consumed from the feeder per dive was recorded.

### Open-flow respirometry

The breathing chamber was connected to an open-flow respirometry system to estimate O_2_ consumption and CO_2_ production. This respirometry setup has been described in detail by Sparling et al. (2008). In short, the breathing chamber had an inlet and an outlet which were both connected to the respirometry system in an adjacent laboratory by flexible 4 cm diameter hosing. Ambient air or a specified gas mix was delivered through the inlet at a constant flow rate. Excurrent air from the breathing chamber was subsampled and drawn through a drying column to two independent sets of analysers measuring the fractional concentrations of O_2_ and CO_2_: FC-10a and Ca-2A analysers from the Sable Data acquisition system (Sable Systems International, Las Vegas, NV, USA), and Fathom Systems' Paramagnetic Oxygen Analyser, iCO2A high-range Analyser and iCO2A low-range Analyser (JFD). The fractional concentrations, pressure, humidity and air temperature were recorded every 1 s.

The volume of oxygen consumed (*V*_O_2__) and volume of CO_2_ produced (*V*_CO_2__) were calibrated using the one-step N_2_-dilution method (Fedak et al., 1981) and CO_2_ addition calibrations, respectively. The following equations for *V*_O_2__ and *V*_CO_2__ were previously presented in McKnight et al. (2025).

*V*_O_2__ (l) was calculated using the following equation:
(1)

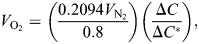
where Δ*C* is the deflection of the analyser during measurement, ΔC* is the deflection of the analyser during calibration and *V*_N_2__ is the volume (l) of nitrogen used in the calibration.

*V*_CO_2__ (l) was calculated using the following equation:
(2)

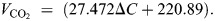
*V*_O_2__ and *V*_CO_2__ were extracted from the beginning of the first dive to the end of the post-diving recovery period to capture metabolic rate across the entire diving trial. For this reason, respirometry extractions were only completed for half of the diving trials that included the post-diving trial recovery period. For each of these diving trials, the mass-specific oxygen consumption rate (*V̇*_O_2__) and mass-specific CO_2_ production rate (*V̇*_CO_2__) were estimated by dividing the *V*_O_2__ and *V*_CO_2__, respectively, by animal mass (kg) and trial duration (min).

### Energy gain

Energy gain (i.e. net rate of energy gain in kJ kg^−1^ min^−1^) was estimated for the same subset of trials by dividing the difference in energy intake and energy expenditure (kJ) by animal mass (kg) and trial duration (min). Energy intake was estimated as the product of (a) the number of sprat consumed per diving trial, (b) the mean wet mass (kg) of sprat and (c) the mean wet mass energy density (kJ kg^−1^) of sprat. The mean wet mass energy density values were provided for a subset of sprat fed to the seals during the diving experiments using bomb calorimetry completed in-house at the Sea Mammal Research Unit. Energy expenditure was calculated by multiplying the *V*_O_2__ of each diving trial by an energy equivalent (kJ *V*_O_2__^−1^) derived from the Weir equation (Weir, 1949) using a respiratory quotient (RQ) of 0.76 (average RQ of grey seals in Boily and Lavigne, 1995; Sparling et al., 2008).

### Blood sampling

Three days prior to the start of an experimental week, participant seals were manually restrained and either pre-medicated with 0.1 mg kg^−1^ of midazolam (Dormazolam, Dechra, Northwich, UK; 5 mg ml^−1^ solution) or sedation was directly induced with a 0.025 mg kg^−1^ of medetomidine hydrochloride (Sedator, Dechra; 1 mg ml^−1^ solution) and 0.1 mg kg^−1^ of butorphanol (Turbogesic, Zoetis UK, Leatherhead, UK; 10 mg ml^−1^ solution) combination. Sedation was maintained with medetomidine hydrochloride and butorphanol, while an intravenous catheter was placed in the extradural intervertebral vein using aseptic technique. After successful venous catheterisation, the catheter was attached to a Bluetooth controlled device, called the ‘Mossquito’, with remote blood sampling and drug administration capabilities, specifically developed for the purpose of this experimental protocol. The Mossquito was attached to the animal using a baseplate that was glued to the fur with Loctite 422 superglue (Henkel UK, Hemel Hempstead, UK), allowing for simple removal and replacement of the device between consecutive use, without removal of the catheter. At the end of an experimental period, the catheter was removed.

Three blood sampling events were repeated across the course of the experimental trials: (1) baseline blood samples were taken after successful catheterisation 3 days before the start of an experimental week; (2) immediately post-diving blood samples were taken within 2 min of a seal returning from its last dive of a diving trial; and (3) 3 h post-diving blood samples were taken approximately 3 h after the end of a diving trial. The immediately post-diving blood samples were collected using the Mossquito, eliminating the need for animal-handling, restraint or chemical immobilisation. These samples were collected twice a week when post-diving blood samples were prioritised over extended respirometry measurements during recovery. On those days, animals were remotely sedated with medetomidine hydrochloride and butorphanol approximately 3 h post-diving, using the Mossquito. The 3 h post-diving samples were collected, and the Mossquito was replaced whilst an animal was sedated. Additionally, ‘true’ baseline blood samples were collected without any physical disturbance using the Mossquito outside of the defined experimental protocol when animals were fully conscious and hauled-out of the water. Following successful instrumentation or de-instrumentation and blood sampling under sedation, sedation was antagonised via intravenous administration of 0.025 mg kg^−1^ of atipamezole hydrochloride (Atipazole, Forte Healthcare, Stamullen, Ireland; 5 mg ml^−1^ solution).

### Blood sample analyses

#### Blood gases and clinical chemistry

A subsample of heparinised blood for each blood sampling point was used to obtain measures of blood gases and clinical chemistry parameters from a handheld blood analysis system (i-STAT 1 analyzer, 04P75-03, Abbott, Abbott Park, IL, USA), previously used in harbour seals (*Phoca vitulina*) (Witte et al., 2014). Following a calibration simulation on the i-STAT system, blood subsamples were added directly to the CG4+ cartridges (03P85-51, Abbott), which were inserted into the i-STAT system for analysis following manufacturer instructions. Although the cartridge provided a broad range of blood gas and clinical chemistry measures, the present study focused on serial measures of lactate (mmol l^−1^).

#### Circulating triglycerides

Circulating triglyceride levels at each blood sampling point were measured using a colorimetric assay for the quantification of triglycerides in plasma, serum, cell lysates and tissue homogenates (Triglyceride Colorimetric Assay Kit, Item No. 10010303, Cayman Chemical, Ann Arbor, MI, USA). The assay standard curve ranges from 0 to 200 mg dl^−1^ with a sensitivity of 0.5 mg dl^−1^. The colorimetric assay was validated for use on grey seal plasma samples by comparing the slopes of the standard curve and concentration-detection relationship of eight plasma samples that were serially diluted at 1:5, 1:10, 1:20 and 1:40 using the standard diluent provided in the assay (i.e. testing for parallelism). The assay demonstrated good parallelism (Fig. S1) with no significant difference between the slope of these dilutions and the standard curve (ANOVA: *F*_1,2_=0.079, *P*=0.806). Based on the results from the parallelism validation test, samples were diluted 1:5 with the standard diluent and were run in duplicate according to the manufacturer's instructions. A total of 11 and 9 samples were assayed for intra- and inter-plate variability, respectively. The mean coefficient of variation was 3.58% for intra-plate variability and 12.0% for inter-plate variability.

### Statistical analyses

Sample size (*n*) was ultimately limited by the number of seals that the Sea Mammal Research Unit Pool Facility could hold and the duration for which they were permitted to be held at the facility. Further, a lack of information on the likely effect sizes associated with the experiment precluded a meaningful *a priori* power analysis; however, the resulting sample size is generally in line with those of previous comparable studies (e.g. Fahlman et al., 2008; Hastie et al., 2021; Hurley and Costa, 2001; Sparling et al., 2007b). All statistical analyses were completed in R Statistical Software (v4.4.1; https://www.r-project.org/). Generalised additive mixed models (GAMMs) and predictions were performed using the ‘mgcv’ package (v1.9.1; Wood, 2011). Generalised linear mixed models (GLMMs) and predictions were performed using the ‘glmmTMB’ package (v1.1.13; Brooks et al., 2017).

#### Impact of gas condition on metabolic rates and energy gain

Analyses were carried out to investigate the influence of different gas conditions on *V̇*_O_2__, *V̇*_CO_2__ and energy gain. Preliminary data exploration indicated that experience days (i.e. days since the start of the first trial for each individual) and sequential day of the week had an impact on all three response variables. The average group and individual responses to gas conditions were modelled using GAMMs. GAMMs were chosen for analysis to capture complex, non-linear relationships between covariates and response variables, and to incorporate random effects, thereby accounting for individual variation. As such, individual was included as a random intercept term, and gas condition was included as a factor term and a factor term with an individual interaction. Experience days and sequential day of the week were included in full models for model selection as global smooth terms and global smooth terms with an individual-level smooth (individual interaction) for the metabolic rate response variables. Sequential day of the week was not included in the full model for energy gain because there was an uneven distribution in sampling days of the week across individuals when *V*_O_2__ and the number of fish consumed per trial were both available for the calculation of energy gain. The distribution family (Gaussian, Gaussian with log link, gamma or gamma with log link) was chosen based on inspection of normality and residual distribution in residual plots. The final model for each response variable was chosen based on backward elimination model selection using Akaike’s information criterion (AIC), with the more parsimonious model being chosen when AIC differed by <2.

#### Impact of blood sampling point on circulating triglyceride concentrations

Circulating triglyceride concentrations were assessed as an indicator of digestive activity. Based on previous work suggesting that grey seals delay digestion following foraging dives (Sparling et al., 2007b), we investigated whether there was evidence of digestive activity both during diving and 3 h post-diving. The 3 h post-diving blood sampling point was employed to represent the peak timing of digestion based on previous findings in juvenile grey seals (Cooper et al., 2005). To investigate whether there was a significant difference in triglyceride concentration at blood sampling points immediately post-diving and 3 h post-diving compared with baseline, triglyceride concentration was modelled against sampling point using a GLMM. All baseline samples (Mossquito and manually collected baseline samples) were included in the model to represent circulating triglyceride concentration in unfed (pre-prandial) animals. Animal ID was included as a random effect to account for individual variation. The distribution family (Gaussian, Gaussian with log link, gamma or gamma with log link) was chosen based on inspection of normality and residual distribution in residual plots.

#### Impact of gas condition on circulating triglyceride concentrations

The effect of gas condition on the timing of digestion was investigated at two distinct time scales. Accordingly, two response variables were included in the analysis for each animal: (1) triglyceride concentration immediately post-diving for each diving trial (TAG), and (2) the difference in triglyceride concentration between samples taken immediately post-diving and 3 h post-diving (ΔTAG). The TAG response term was included in the analysis to assess whether gas condition influenced digestive activity during diving, which may in turn affect energy gain (i.e. short-term effects). The ΔTAG response term was included in the analysis to assess whether gas condition influenced the peak timing of digestion at 3 h post-diving, which does not directly influence energy gain during diving but instead reflects how the overall energy intake is subsequently utilised (i.e. long-term effects). In other words, ΔTAG helps inform on whether other post-diving metabolic processes, such as the clearing of anaerobic byproducts, were prioritised over digestion in certain gas conditions.

The responses in TAG and ΔTAG to gas condition were also modelled using GAMMs. As above, individual was included as a random intercept term and gas condition was included as a factor term and a factor term with an individual interaction. Furthermore, experience days, mass (kg) and time difference between the start of diving and the first blood sample (immediately post-diving) were included as global smooth terms and global smooth terms with an individual-level smooth in the full model undergoing model selection for both response variables. The time difference between the start of diving and the second blood sample (3 h post-diving) was additionally included as a global smooth term and global smooth term with an individual-level smooth in model selection for the response in ΔTAG. Sequential day of the week was not included in the full models because the smaller sample sizes meant that there was an uneven distribution in sampling days of the week across individuals. The full models for TAG and ΔTAG underwent the same backward elimination model selection process as the previously described GAMMs.

#### Impact of gas condition on lactate concentration

To investigate whether gas condition influenced a shift to anaerobic metabolism during diving, lactate concentration at baseline and immediately post-diving was modelled against gas condition using a GLMM with animal ID as a random effect to account for individual variation. Values from Mossquito baseline samples were used to represent ‘true’ baseline lactate concentrations, as lactate concentration was previously demonstrated to be influenced by periods of apnoea in seals under sedation (Kershaw et al., 2026).

*P*-values for the effect of gas condition on energy gain, *V̇*_O_2__, *V̇*_CO_2__, TAG, ΔTAG and lactate concentration, and for the effect of sampling point on triglyceride concentration, were obtained from the fixed-effects estimates of GAMM or GLMM outputs in which each gas condition or sampling point was compared with the model intercept (baseline or ambient). All presented plots were generated using ‘ggplot2’ (v3.5.1; Wickham, 2016).

## RESULTS

A total of 119 diving trials consisting of 510 dives were completed by the six grey seals in this study from November 2023 to March 2024 (see Table S1 for detail on the number of data points for each gas condition and individual). Each seal completed 4–6 diving trials in each gas condition (between 29 and 31 trials in total for each condition; Table S1). Mean values are presented ±s.e.m.

### Energy gain

**Fig. 2.**
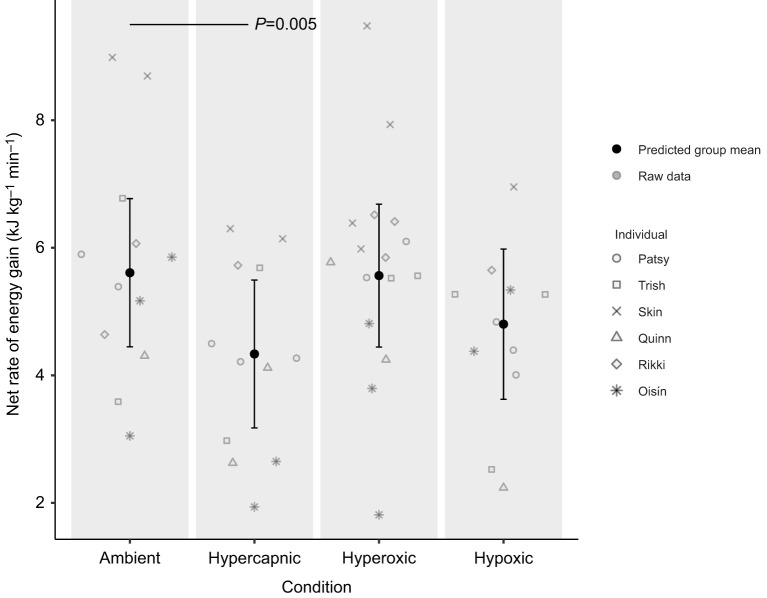
**Predicted group mean and individual raw data points for the response in energy gain (kJ** **kg^−1^** **min^−1^) of six juvenile grey seals (3 females=Patsy, Trish and Skin; 3 males=Quinn, Rikki and Oisín) across ambient (*n*=12), hypercapnic (*n*=12), hyperoxic (*n*=16) and hypoxic (*n*=11) gas conditions.** Error bars indicate 95% confidence intervals. The solid black horizontal line indicates a significant difference from ambient with the associated *P*-value. Predicted group mean values were obtained from model predictions generated by a generalised additive mixed model (GAMM). Energy gain, net rate of energy gain.

The energy gain was calculated for 51 diving trials (Table S1). The predicted group mean and individual raw data points of energy gain for each gas condition are presented in Fig. 2 (see Tables S2 and S3 for model details). Energy gain decreased significantly under hypercapnia compared with ambient conditions (mean 4.26±0.43 and 5.70±0.52 kJ kg^−1^ min^−1^, respectively, *P*=0.005). There was no significant difference in energy gain under hyperoxic (mean 5.73±0.42 kJ kg^−1^ min^−1^, *P*=0.912) and hypoxic (mean 4.62±0.41 kJ kg^−1^ min^−1^, *P*=0.077) conditions compared with ambient.

### Metabolic rate

**Fig. 3.**
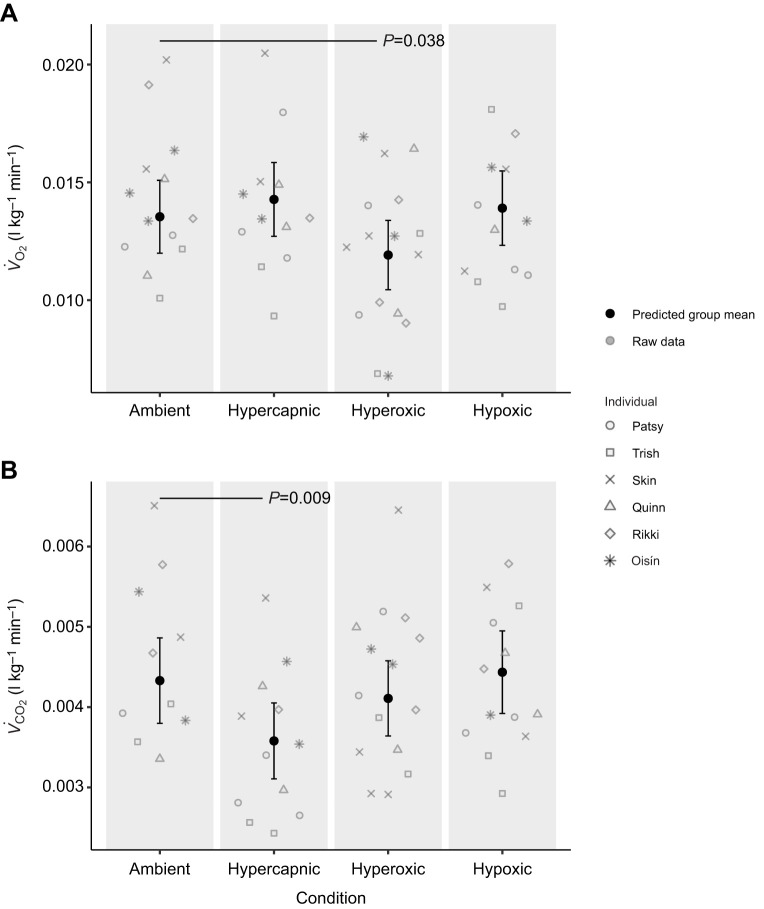
**Predicted group mean and individual raw data points for the responses in mass-specific rates of oxygen consumption and carbon dioxide production.** Data are shown for (A) *V̇*_O_2__ (l kg^−1^min^−1^) and (B) *V̇*_CO_2__ (l kg^−1^min^−1^) of six juvenile grey seals (3 females=Patsy, Trish and Skin; 3 males=Quinn, Rikki and Oisín) across gas conditions. Sample size for *V̇*_O_2__ in each condition: ambient *n*=13, hypercapnic *n*=12, hyperoxic *n*=16 and hypoxic *n*=12. Sample size for *V̇*_CO_2__ in each condition: ambient *n*=10, hypercapnic *n*=12, hyperoxic *n*=15 and hypoxic *n*=13. Error bars indicate 95% confidence intervals. Solid black horizontal lines indicate significant differences from ambient with associated *P*-values. Predicted group mean values were obtained from model predictions generated by GAMMs. *V̇*_O_2__, mass-specific rate of oxygen consumption; *V̇*_CO_2__, mass-specific rate of carbon dioxide production.

*V̇*_O_2__ and *V̇*_CO_2__ were calculated across 53 and 50 diving trials, respectively (Table S1). The predicted group mean and individual raw data points of *V̇*_O_2__ and *V̇*_CO_2__ for each gas condition are presented in Fig. 3 (see Tables S2, S4 and S5 for model details). There was a significant decrease in *V̇*_O_2__ under hyperoxia compared with ambient conditions (mean 0.012±0.0008 and 0.014±0.0008 l kg^−1^ min^−1^, respectively, *P*=0.038). There was no significant change in *V̇*_O_2__ under hypercapnia (mean 0.014±0.0009 l kg^−1^ min^−1^, *P*=0.368) and hypoxia (mean 0.013±0.0008 l kg^−1^ min^−1^, *P*=0.657) compared with ambient conditions.

*V̇*_CO_2__ decreased significantly under hypercapnia (mean 0.0035±0.0003 l kg^−1^ min^−1^, *P*=0.009). There was no significant difference in *V̇*_CO_2__ under hyperoxic (mean 0.0043±0.0003 l kg^−1^ min^−1^, *P*=0.384) and hypoxic (mean 0.0043±0.0002 l kg^−1^ min^−1^, *P*=0.702) conditions compared with ambient (mean 0.0046±0.0003 l kg^−1^ min^−1^).

### Circulating triglycerides: effect of sampling point

**Fig. 4.**
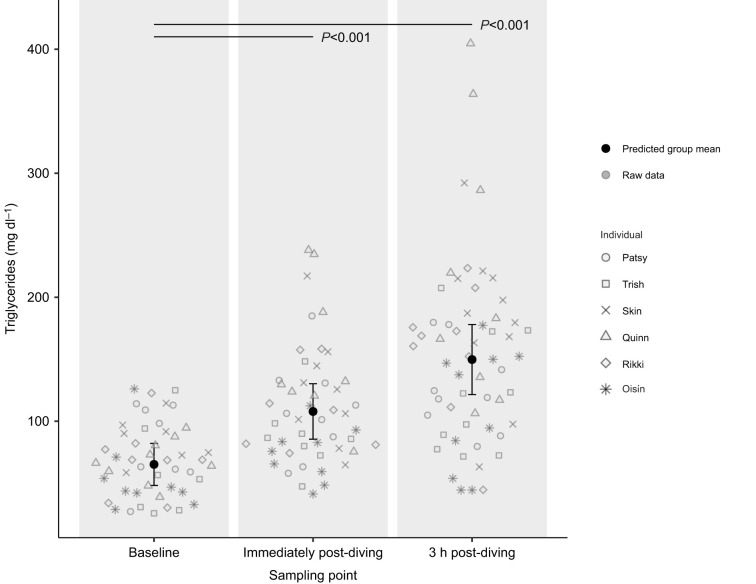
**Predicted group mean and individual raw data points for circulating triglyceride concentration (mg** **dl^−1^) of six juvenile grey seals (3 females=Patsy, Trish and Skin; 3 males=Quinn, Rikki and Oisín) at baseline (*n* =48), immediately post-diving (*n*=50) and 3 h post-diving (*n*=58).** Error bars indicate 95% confidence intervals. Solid black horizontal lines indicate significant differences from baseline with associated *P*-values. Predicted group mean values were obtained from model predictions generated by a generalised linear mixed model (GLMM).

Triglyceride concentration was modelled across 48 baseline samples, 50 samples for immediately post-diving and 58 samples for 3 h post-diving (Table S1). There was one less sample for triglyceride concentration at immediately post-diving than for lactate concentration owing to one sampling event in which not enough blood was collected to store for analyses. The predicted group mean and individual raw data points of triglyceride concentration for each sampling point are presented in Fig. 4 (see Tables S6 and S7 for model details). There was a significant increase in triglyceride concentration immediately post-diving (mean 109.9±6.51 mg dl^−1^, *P*<0.001) and 3 h post-diving (mean 152.2±9.45 mg dl^−1^, *P*<0.001) compared with baseline (mean 69.0±4.15 mg dl^−1^).

### Circulating triglycerides: effect of gas condition

The response in triglyceride concentration immediately post-diving (TAG) was measured for 50 trials (Table S1). The GAMM output showed no significant effect of gas condition on TAG (*P*=0.501; see Tables S2 and S8 for model details).

The response in the change in triglyceride concentration from immediately post-diving to 3 h post-diving (ΔTAG) was measured for 49 trials for which blood samples at both sampling points were collected (Table S1). As for the response in TAG, the resulting GAMM also showed no significant effect of gas condition on ΔTAG (*P*=0.318; see Tables S2 and S9 for model details).

### Lactate

**Fig. 5.**
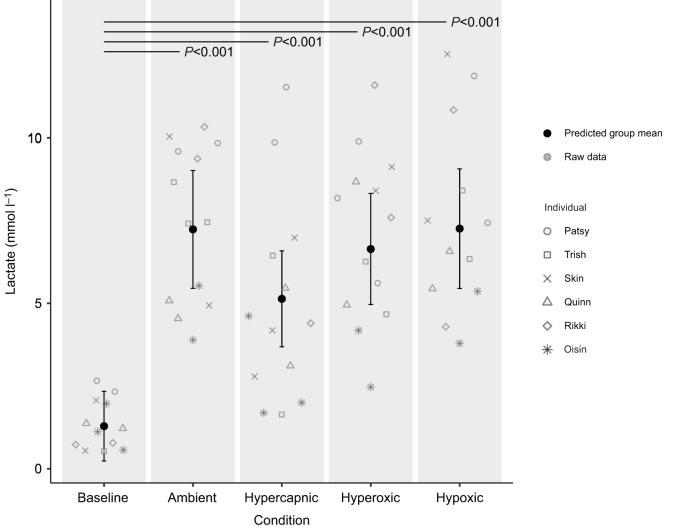
**Predicted group mean and individual raw data points for lactate concentration (mmol** **l^−1^) in six juvenile grey seals (3 females=Patsy, Trish and Skin; 3 males=Quinn, Rikki and Oisín) at baseline (Mossquito baseline, *n*=12) and immediately post-diving across ambient (*n*=13), hypercapnic (*n*=13), hyperoxic (*n*=13) and hypoxic (*n*=12) gas conditions.** Error bars indicate 95% confidence intervals. Solid black horizontal lines indicate significant differences from baseline with associated *P*-values. Predicted group mean values were obtained from model predictions generated by a GLMM.

Lactate concentration was measured for 12 baseline blood samples and 51 blood samples taken immediately post-diving (Table S1). The predicted group mean and individual raw data points of lactate concentration at baseline and for each gas condition are presented in Fig. 5 (see Tables S6 and S10 for model details). There was a significant increase in lactate immediately post-diving across all gas conditions compared with baseline (mean baseline 1.3±0.2 mmol l^−1^; ambient 7.4±0.7 mmol l^−1^; hypercapnic 5.0±0.9 mmol l^−1^; hyperoxic 7.0±0.7 mmol l^−1^; hypoxic 7.5±0.8 mmol l^−1^; all *P*<0.001).

## DISCUSSION

In the present study, six grey seals were exposed to one of four respiratory gas conditions (ambient, hypercapnic, hypoxic and hyperoxic) during surface intervals in a simulated foraging setup. The effect of gas condition on energy gain and underlying changes in metabolic rate, timing of digestion and post-dive lactate concentration were investigated and presented in Figs 2–5. The variability observed in the raw data points across Figs 2–5 may be attributed to both within individual variation owing to ontogenetic changes over the experimental period, and between-individual variation owing to differences in diving abilities or decision-making.

To our knowledge, this is the first study to quantify the effect of changes in circulating O_2_ and CO_2_ on energy gain in freely diving seals. Using empirical data, our findings confirm the hypotheses in previous studies (Fahlman et al., 2008; Gerlinsky et al., 2014) that the accumulation of CO_2_ affecting surface interval duration will decrease energy gain by limiting the relative amount of time spent diving. Furthermore, we present evidence of digestion and anaerobic metabolism in grey seals completing short intensive diving bouts, which contrasts with previous theories of optimal foraging behaviour suggesting delayed digestion and diving within the aerobic dive limit.

### Energy gain

Phocid seals, like other marine mammals, are limited in their foraging efficiency by the requirement to surface between dives. To maximise their energy gain during foraging bouts, they should therefore seek to minimise the time spent at the surface and maximise the time spent diving. This balance depends on a number of intrinsic and extrinsic factors. Extrinsic factors that may influence dive behaviour include prey patch density, distance from prey patch to the surface, predation risk and time spent searching for prey (Heithaus and Frid, 2003; Sparling et al., 2007a; Thompson and Fedak, 2001). In the present study, we sought to control these extrinsic factors by standardizing a 60-m transit to the underwater feeder and delivering a set amount of fish (1.5 kg of sprat) at a constant rate, thereby simulating a foraging patch of fixed prey density across all diving trials and gas conditions. Additionally, a single prey species (i.e. sprat) was chosen for the diving trials to standardise prey energy density, thereby controlling for variation in prey quality and its effect on energy gain.

The measures of energy gain observed in this study fall within the range previously reported for pups and adult grey seals housed in the same facility, where animals were fed a diet of herring and sandeels and dived to three different target distances (Sparling et al., 2007a). Although there was no significant change in energy gain under hypoxia, hypercapnia caused a significant decrease in energy gain (Fig. 2). Given that metabolic rate (*V̇*_O_2__) and changes in circulating triglyceride levels (TAG) were not significantly influenced by hypercapnia, the change in energy gain under hypercapnia was likely driven by the increased IDSI duration (McKnight et al., 2025). This finding supports a previous study suggesting that the increased time required for CO_2_ removal extends the IDSI, thereby limiting the proportion of time spent diving; a cost that ultimately limits foraging efficiency (Gerlinsky et al., 2014).

Other factors that may have affected energy gain, which were not explored in the present study, may include physiological mechanisms to cope with inflammation and oxidative stress associated with hypoxemia and reduced peripheral perfusion in diving mammals (Allen and Vázquez-Medina, 2019; Vázquez-Medina et al., 2012). For example, higher activity of antioxidant enzymes has been reported in diving versus non-diving birds and mammals, including phocid seals (Allen and Vázquez-Medina, 2019). The anti-inflammatory and antioxidant strategies employed by seals to manage hypoxemia and ischemia–reperfusion injury may have been used differentially across gas conditions.

### Dive behaviour

As reported in McKnight et al. (2025), all seals in the present study showed no apparent aversion to the hypercapnic condition and continued diving despite a 200-fold increase in CO_2_ compared with ambient levels, with no significant effect of hypercapnia on dive duration. Similarly, Gerlinsky et al. (2014) found no significant effect of hypercapnia (up to 3% CO_2_) on dive duration in freely diving Steller sea lions (*Eumetopias jubatus*). Hypoxia and hypercapnia both significantly increased IDSI duration compared with the ambient condition (McKnight et al., 2025). The effect of increased circulating CO_2_ on IDSI duration supports findings from previous studies suggesting that the accumulation of CO_2_ across repeated dives with short surface intervals necessitates a prolonged final surface interval to eliminate CO_2_ through exhalation (Boutilier et al., 2001; Fahlman et al., 2008; Falke et al., 2008; Gerlinsky et al., 2014; Kooyman et al., 1973; Rosen et al., 2017). As previously mentioned, this phenomenon likely reflects the slower rate of CO_2_ elimination compared with O_2_ replenishment, owing to the additional biochemical step of converting bicarbonate and H^+^ back to CO_2_ for exhalation.

### Metabolic rate

For the purpose of this study, metabolic cost was estimated for complete diving bouts, as single-dive estimates of *V̇*_O_2__ and *V̇*_CO_2__ likely do not account for potential O_2_ debt or CO_2_ accumulation from preceding dives (Fahlman et al., 2008; McKnight et al., 2019). Inclusion of the extended post-diving recovery periods in the present study was made to account for these potential cumulative effects and differed from the analysis presented in McKnight et al. (2025) in which *V̇*_O_2__ and *V̇*_CO_2__ were estimated across diving bouts without the inclusion of the recovery period. The predicted changes in *V̇*_O_2__ across gas condition from the GAMM suggest that there was no significant effect of hypoxia and hypercapnia on metabolic rate of O_2_ but that seals responded to hyperoxia with hypometabolism compared with ambient levels (Fig. 3). The significant decrease in *V̇*_O_2__ under hyperoxia likely supported longer dive durations observed under this condition by conserving O_2_ (McKnight et al., 2025). The absence of an associated significant reduction in *V̇*_CO_2__ under hyperoxia suggests that animals were saturated with O_2_, thereby requiring greater CO_2_ offloading relative to O_2_ uptake. It also may suggest that the rate of CO_2_ offloading is limited, potentially owing to a lack of plasticity in respiration rate (see ‘Lactate’; Bliault, 2025).

Whilst hyperoxia resulted in a significant decrease in *V̇*_O_2__, hypercapnia caused a significant decrease in *V̇*_CO_2__ compared with the ambient condition (Fig. 3). The significant decrease in *V̇*_CO_2__ under hypercapnia may be explained by CO_2_ retention: a lack of CO_2_ diffusion between the lungs and mixed venous blood because the seals' blood gas CO_2_ tensions may be similar or equal to those of the breathing chamber (i.e. 8%). As such, the significant influence of hypercapnia on *V̇*_CO_2__ is likely due to the experimental setup and may not reflect physiologically relevant conditions in natural diving scenarios.

The calculation of energy gain relied on an RQ value (*V*_CO_2__/*V*_O_2__) to derive an energy equivalent: the amount of energy (in kJ) released per litre of O_2_ consumed. In this scenario, the RQ reflects which substrates were being metabolised. Under resting metabolic conditions, the respiratory exchange ratio (RER; *V*_CO_2__/*V*_O_2__) measured at the lungs can be used as a proxy for the RQ at the cellular level. Owing to the effect of CO_2_ retention under hypercapnia, it was not possible to reliably calculate the RER for diving trials in this condition, and therefore comparisons in RER could not be made across all gas conditions. Furthermore, the RER values were generally low across all diving trials with a mean of 0.31±0.01 and range of 0.19–0.52. These values are below the theoretical range associated with substrate metabolism (∼0.7–1.0). Given the rapid rate of O_2_ loading in phocid seals during relatively short surface intervals (Meir et al., 2009; Ponganis et al., 1993), we suggest that the low RER values were a result of incomplete CO_2_ offloading during this intensive diving scenario, even with the extended 5-min recovery period. It was due to these effects that an average RER value from previous grey seal literature was used in the calculation of energy gain across all diving trials. We do not expect the ‘true’ RQ/RER values (with complete CO_2_ offloading) to have differed significantly between gas conditions given that animals were given the same diet, were the same age class, completed diving trials at the same time of day, and completed relatively short diving bouts (17–46 min).

### Circulating triglycerides: effect of sampling point

The initial step in the analysis of circulating triglycerides was to determine whether digestion was evident both immediately post-diving and 3 h post-diving. Previous work on postprandial circulating triglycerides and lipoproteins in juvenile grey seals showed that concentrations peaked at 3 h post-feeding (Cooper et al., 2005). Thus, the 3-h post-diving blood sampling point used in the experiments for this study was employed to capture the effect of respiratory gas condition on the timing of digestion. Indeed, there was a significant increase in circulating triglycerides 3 h post-diving compared with baseline (Fig. 4), although the extent to which we captured the actual peak timing of digestion remains unclear and is discussed below (see ‘Circulating triglycerides: effect of gas condition’).

Notably, there was also a significant increase in circulating triglycerides immediately post-diving, which is indicative of digestion during diving. Although forced diving experiments indicated blood restriction to splanchnic organs (Zapol et al., 1979), which is likely representative of extreme haemodynamic responses, findings from freely diving animals are more variable. Foraging scenarios in the wild are dynamic, with several diving bouts occurring in a day over 3–10 day foraging trips (McConnell et al., 1999; Thompson et al., 1991). The benefit of digesting during diving, as observed in the present study, would be the continuous energy uptake to support further diving, but this competes with O_2_ demands of the muscles, heart and brain, potentially reducing dive durations. Delaying digestion could preserve O_2_ to maximise diving abilities and may be more advantageous when seals encounter high quality prey patches.

Our results show that voluntarily diving juvenile grey seals can process food even during energetically demanding dives; seals in our study were not only diving in high circulating CO_2_ and low circulating O_2_ conditions, but also incurred a significant build-up of circulating lactate after diving in all gas conditions, including ambient (Fig. 5). Although the present study is not representative of repeated diving bouts in a patchy environment, blood samples taken from Weddell seals (*Leptonychotes weddellii*) completing multiple foraging dives over several hours also indicate ongoing digestion and elevated post-dive lactate concentrations (Castellini et al., 1988; Davis et al., 1983). In contrast, studies suggesting delayed digestion in grey seals and Steller sea lions have relied on respirometry-based measures of metabolic rate as opposed to measuring direct markers of digestion (Rosen et al., 2015; Sparling et al., 2007b). This highlights the need for caution when interpreting post-dive increases in metabolic rate solely as digestion, as other physiological factors may contribute.

It should be noted that in the current study seals engaged in a single, relatively short, predictable foraging bout each day and were free to rest for the remainder of the day. The daily food ration of 1.5 kg sprat and 0.5 kg herring during diving trials in the present study was determined to maintain the seals' motivation and mass across the experimental period. This daily intake represents just over half of the estimated energy requirements for the ‘average’ grey seal in the UK (∼25,300 kJ day^−1^) (Sparling and Smout, 2003), and similarly exceeds half of the average daily fish intake by pups and sub-adults in France and the UK during October–March (∼2.55 kg day^−1^) (Vincent et al., 2016). Given that the diving trials in the present study represented the sole foraging event of the day and that the seals may not have reached full satiety during experiment weeks, they may have engaged in more energetically demanding dives than would typically occur under natural foraging conditions.

### Circulating triglycerides: effect of gas condition

The second step of the analysis in circulating triglyceride levels was to determine the effect of gas condition on digestive activity at two different time scales: immediately post-diving (TAG) and 3 h post-diving at the expected peak timing of digestion (ΔTAG). Given the added challenge of diving under hypercapnia or hypoxia, we hypothesised that animals would prioritise gas regulation and the clearing of potential anaerobic metabolites over digestion, thereby delaying digestion under these conditions. However, neither TAG nor ΔTAG were significantly influenced by gas condition. This suggests that seals do not prioritise gas regulation or the clearing of potential anaerobic metabolites over digestion.

It is important to consider that despite the findings from Cooper et al. (2005), the 3-h sampling window used in our study may not have captured the peak timing of digestion and, therefore, ΔTAG was not representative of the expected peak change in triglyceride concentration from diving. The peak timing of digestion in the present study may have differed from that of Cooper et al. (2005) owing to several aspects of our methodology, including: (1) the comparatively lower food intake of grey seals during experimental trials; (2) seals had to actively dive to acquire food; (3) a remote blood sampler, the ‘Mossquito’, was used to collect post-diving blood samples, meaning digestion was not influenced by the acute stress response related to repeated capture and handling (e.g. Champagne et al., 2012b; Cooley et al., 2025; Takei et al., 2016); and (4) seals were free to haul-out or swim in non-experimental pool areas between the end of a diving trial and the 3-h post-diving blood sampling, meaning the timing of peak digestion may have differed between individuals or trial days depending on the animals' activity levels prior to sampling at 3 h post-diving.

The colorimetric assay using for the quantification of circulating triglyceride concentration isolated glycerol as a proxy for triglyceride content. In fasting pinnipeds, high rates of lipolysis in blubber increase circulating glycerol levels compared with the non-fasting state. A small fraction of the circulating glycerol is then used for gluconeogenesis to synthesise glucose for tissues that cannot rely solely on fat for energy (e.g. the brain) (Champagne et al., 2012a). Similarly, during diving, an increase in circulating glycerol concentration in harbour seals has been attributed to continued lipolysis (Davis, 1983). However, this response may have been influenced by the heightened hormonal stress response associated with forced diving scenarios in those experiments (Davis, 1983; Hance et al., 1982). Thus, the extent to which freely diving seals rely on lipolysis of fat stores and mobilisation for gluconeogenesis is unclear, but ultimately may have influenced the levels of glycerol collected immediately post-diving. Therefore, we may have measured a combination of both digestion and lipolysis. A useful avenue for future research may be the incorporation of digestion-specific markers of postprandial fat absorption, such as chylomicron lipoprotein concentration, to better isolate effects from other lipid metabolism processes.

### Lactate

According to optimal foraging theories, shallow divers such as grey seals should maximise net energy gain by performing short, aerobic dives that avoid the accumulation of anaerobic byproducts such as lactate, thereby minimising surface interval duration (Sparling et al., 2007a; Thompson and Fedak, 1993, 2001). The aerobic dive limit (ADL) is the maximum dive duration within which circulating lactate levels do not increase above resting baseline levels, meaning animals rely on aerobic metabolism throughout the dive (Kooyman et al., 1983). In the absence of blood lactate measurements, the ADL has been calculated (cADL) by dividing an animal's estimated total volume of O_2_ stores (l) by its metabolic rate (*V*_O_2__; l min^−1^). To date, optimal foraging studies on grey seals have relied on cADLs, and concluded that grey seals rarely exceed their ADL even at high density prey patches (Sparling et al., 2007a; Thompson and Fedak, 1993, 2001). However, in the present study we found that, at least for juvenile grey seals, a significant increase in blood lactate levels following diving in all gas conditions, including ambient, does occur during short intensive diving bouts (Fig. 5).

Critically, these findings suggest that (1) the accumulation of lactate did not limit dive duration in grey seals under the simulated foraging conditions of this study, and (2) in the short term, the clearance of anaerobic metabolic byproducts did not significantly contribute to the reduced energy gain under hypercapnia. Ultimately, the accumulation of CO_2_ and its slow rate of removal was a greater limiting factor to foraging efficiency than the aerobic dive limit. This supports the concept that, if O_2_ is the main driver of dive duration, surfacing with a greater O_2_ debt allows animals to load O_2_ more rapidly owing to the higher haemoglobin affinity at lower partial pressures (Fahlman et al., 2008; McKnight et al., 2025). Therefore, in terms of optimal foraging strategy, it may be more efficient, at least in the short term, to prioritise replenishing O_2_ at the steep part of the O_2_ dissociation curve, where small increases in partial pressures of O_2_ result in large increases in haemoglobin saturation, rather than to avoid lactate accumulation. In other words, the accumulation of lactate owing to anaerobic metabolism under decreasing O_2_ tensions is a tolerable short-term trade-off to rapidly reload O_2_ at the surface because evidently O_2_, and not lactate, is the main limiting factor to dive duration.

Lactate clearance could potentially be facilitated by an elevated respiration rate, which would increase O_2_ availability for the conversion of lactate into pyruvate for use in gluconeogenesis. However, in an independent study from the same experimental trials presented in the present study, the ventilatory response across gas conditions was investigated and respiration rate did not change (Bliault, 2025). This means that neither lactate clearance nor CO_2_ removal were supported by an increase in respiration rate under hypercapnia, a response previously reported in gas manipulation studies on marine mammals (Gallivan, 1980; Gerlinsky et al., 2014; Kohin et al., 1999; McCormick, 1969; Milsom et al., 1996; Parkos and Wahrenbrock, 1987; Påsche, 1976; Skinner and Milsom, 2004). The lack of change in respiratory frequency across gas conditions suggests that grey seals may have an attenuated physiological capacity to modulate respiration rate in response to changes in circulating gas tensions as a result of diving; this further supports the conclusion that the accumulation of CO_2_ is a primary factor driving surface behavioural changes in response to circulating gas conditions and, in turn, limiting foraging efficiency.

### Practical implications

The percentage of inspired O_2_ for the hypoxic condition and CO_2_ for the hypercapnic condition were within physiological thresholds of circulating and end-tidal gas concentrations previously reported in diving phocid seals. End-tidal CO_2_ concentrations of up to 8–9% have been reported during surface intervals of freely diving Weddell seals (Falke et al., 2008). Equally, arterial partial pressures of O_2_ between 12 and 23 mmHg have been recorded in freely diving Weddell seals and northern elephant seals (*Mirounga angustirostris*), equating to approximately 9–11% of inspired O_2_, according to the alveolar gas equation (assuming alveolar partial pressure=arterial partial pressure; Meir et al., 2009; Qvist et al., 1986). Given that O_2_ availability limits dive duration (McKnight et al., 2025), it is possible that a greater level of hypoxia than used in the present study may have a significant effect on the net rate of energy gain. However, within the limits of naturally occurring circulating gas tensions explored in our study, the accumulation of CO_2_ was a greater limiting factor to the net rate of energy gain than the depletion of O_2_.

From an applied perspective, the findings from this study can help inform how foraging behaviour and the resulting energy gain of seals are affected by environmental stressors in the wild. Importantly, the accentuated dive response of seals has some level of premeditation (i.e. cortico-hypothalamic mediation), allowing them to match the degree of the dive response to the expected challenge of a dive (Jones et al., 1973; McKnight et al., 2019; Thompson and Fedak, 1993). As such, seals have some control over the regulation of absolute and time-dependent magnitudes of change in blood gases (i.e. O_2_ and CO_2_) and metabolic byproducts (i.e. lactate). However, this premeditated physiological control may be compromised by behavioural responses to unpredicted disturbance events. For example, repeated disturbance events may cause an animal to terminate any given surface interval early and extend their dives, thereby accumulating CO_2_ and necessitating a much longer surface interval at the end of a disturbance event, ultimately affecting net energy gain. Previous work in narwhals (*Monodon monoceros*) demonstrated that disturbance caused a mismatch between O_2_ demands associated with increased swimming effort and the necessary cardiovascular adjustments (i.e. increased heart rate and muscle perfusion) (Williams et al., 2017, 2022). As a result, narwhals were predicted to deplete the majority of their O_2_ stores, likely causing hypoxemia and hypercapnia, and increasing the energetic cost of diving. These findings highlight the importance of considering physiological constraints that may limit the ability of animals to respond to disrupted foraging and compensate for periods of reduced energy gain.

### Conclusions

The manipulation of inhaled respiratory gas exposures has previously been employed to study sensitivities to different O_2_ and CO_2_ concentrations, and gas management strategies of diving animals. In our study, this method was applied in a simulated foraging setup to quantify how changes in circulating gas tensions of O_2_ and CO_2_ affect metabolic rate, post-dive lactate concentration, the timing of digestion, and energy gain in voluntarily diving grey seals. Exposure to hypercapnia (high CO_2_) significantly decreased energy gain compared with ambient conditions. This effect was attributable to the associated prolonged surface interval durations under hypercapnia. Dive duration, respirometry-derived metabolic rate and the timing of digestion were investigated as other potential factors driving changes in energy gain but had no significant effect under hypercapnia compared with ambient conditions. Post-dive lactate concentration increased significantly after diving in all gas conditions, showing that in isolation, the processing of anaerobic byproducts did not have a significant effect on the decline in energy gain under hypercapnia. The increased surface interval duration under hypercapnia was likely due to the increased recovery times required to offload CO_2_. The consequence of these increased recovery times at the surface was less time spent diving (i.e. foraging), showing that the accumulation of CO_2_ over a diving bout may be an important limiting factor in optimal foraging strategies. As marine mammals have specialised physiological adaptations to endure long periods of diving apnoea and regulate behavioural and energetic efficiency, any changes in physiological regulation may have detrimental effects on their ability to dive, forage and recover. As such, understanding the effect of different circulating gas tensions on energy gain is important for understanding how free-ranging seals may respond to environmental change or disturbance.

## Supplementary Material

10.1242/jexbio.251718_sup1 Supplementary information
