# Supplementary material for: Accumulation of CO2 limits energy gain in freely diving grey seals
Source: J Exp Biol. 2026 May 8;229(9):jeb251718. doi: 10.1242/jeb.251718 (PMC13200726; doi:10.1242/jeb.251718)
Supplement: Supplementary information [file jexbio-229-251718-s1.pdf]

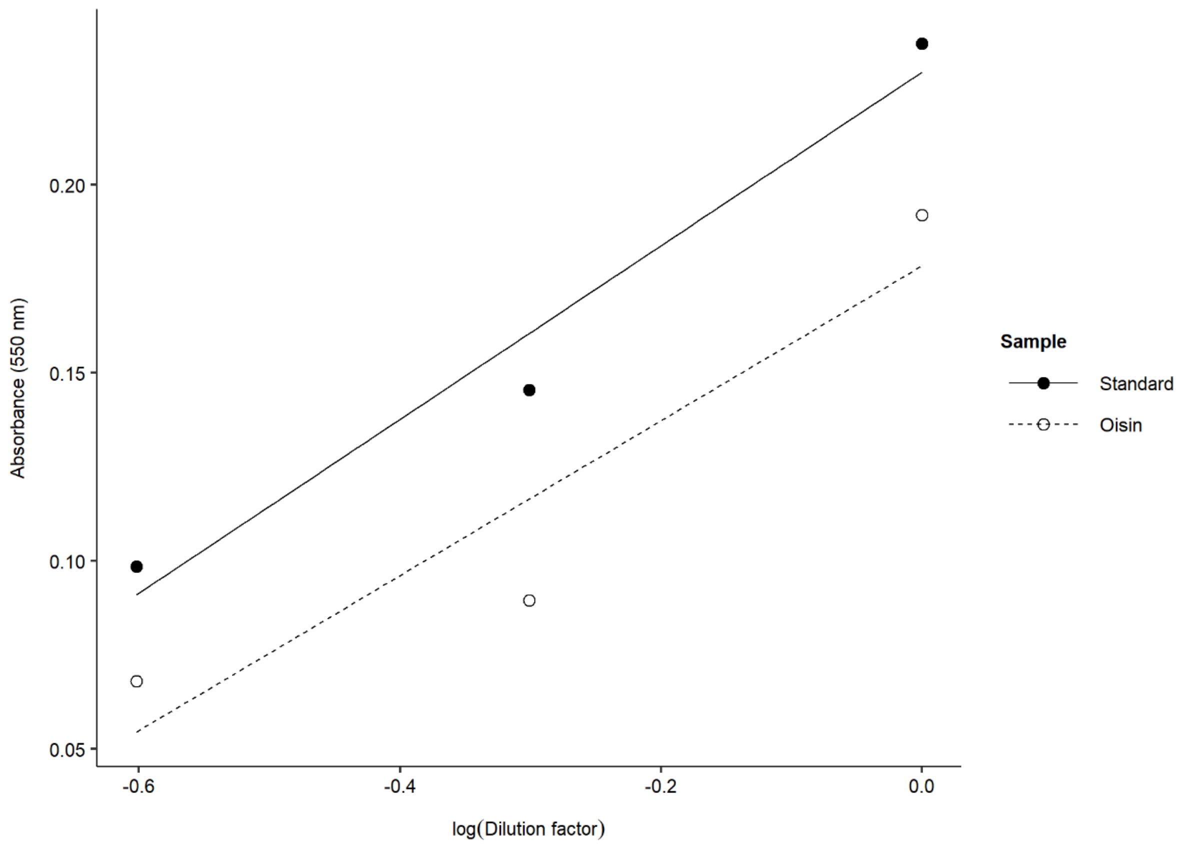

Fig. S1. Validation results for plasma sample dilutions of Oisín. Dashed lines represent best-fit linear regression lines and demonstrate close parallelism between serially diluted samples and the standard curve.

Table S1. The number of diving trials, dives, $\dot{V}O_2$, $\dot{V}CO_2$, net rate of energy gain values, immediately post-diving blood samples (within 2 min post-diving), 3 hrs post-diving blood samples, and ΔTAG values collected per animal and gas condition that were included in the analysis. $\dot{V}O_2$, mass-specific rate of oxygen consumption; $\dot{V}CO_2$, mass-specific rate of carbon dioxide production; ΔTAG, change in triglyceride concentration between immediately post-diving and 3 hrs post-diving.

**Ambient**								
**Animal**	**Trials**	**Dives**	**$\dot{V}O_2$**	**$\dot{V}CO_2$**	**Net energy gain**	**Immediate blood sample**	**3 hrs blood sample**	**ΔTAG**
Patsy	5	22	2	1	2	2	2	2
Quinn	5	28	2	1	1	2	2	1

Journal of Experimental Biology • Supplementary information

Trish	5	12	2	2	2	3	3	3
Rikki	5	16	2	2	2	2	3	2
Skin	6	33	2	2	2	2	4	2
Oisín	5	23	3	2	3	2	2	2
**Total**	31	134	13	10	12	13	16	12
**Hypercapnic**								
**Animal**	**Trials**	**Dives**	**$\dot{V}O_2$**	**$\dot{V}CO_2$**	**Net energy gain**	**Immediate blood sample**	**3 hrs blood sample**	**ΔTAG**
Patsy	5	22	3	3	3	2	2	2
Quinn	4	17	2	2	2	2	2	2
Trish	5	13	2	2	2	2*	2	1
Rikki	5	14	1	1	1	1	2	1
Skin	5	29	2	2	2	3	3	3
Oisín	5	22	2	2	2	3	3	3
**Total**	29	117	12	12	12	13	14	12
**Hyperoxic**								
**Animal**	**Trials**	**Dives**	**$\dot{V}O_2$**	**$\dot{V}CO_2$**	**Net energy gain**	**Immediate blood sample**	**3 hrs blood sample**	**ΔTAG**
Patsy	5	17	2	2	2	3	3	3
Quinn	4	21	2	2	2	2	2	2
Trish	5	14	2	2	2	2	3	2
Rikki	5	13	3	3	3	2	2	2
Skin	6	25	4	4	4	2	2	2
Oisín	5	25	3	2	3	2	2	2
**Total**	30	115	16	15	16	13	14	13
**Hypoxic**								
**Animal**	**Trials**	**Dives**	**$\dot{V}O_2$**	**$\dot{V}CO_2$**	**Net energy gain**	**Immediate blood sample**	**3 hrs blood sample**	**ΔTAG**
Patsy	5	26	3	3	3	2	2	2
Quinn	5	26	1	2	1	2	3	2

Journal of Experimental Biology • Supplementary information

Trish	5	15	3	3	3	2	2	2
Rikki	4	13	1	2	1	2	2	2
Skin	5	30	2	2	1	2	2	2
Oisín	5	34	2	1	2	2	3	2
**Total**	29	144	12	13	11	12	14	12

*For one of Trish's blood samples taken immediately after diving under hypercapnia there was enough blood to get a value for lactate concentration but not for the quantification of triglycerides.

Table S2. Model information of GAMMs for the response in net rate of energy gain (kJ kg^{-1}min^{-1}), $\dot{V}O_2$ (L kg^{-1}min^{-1}), $\dot{V}CO_2$ (L kg^{-1}min^{-1}), TAG (mg dL^{-1}), and ΔTAG (mg dL^{-1}) across gas conditions. The AIC, ΔAIC (full model AIC – final model AIC), adjusted R^2, distribution family, number of diving trials (*n*), and deviance explained (%) are presented for final models. GAMM, generalised additive mixed model; $\dot{V}O_2$, mass-specific rate of oxygen consumption; $\dot{V}CO_2$, mass-specific rate of carbon dioxide production; TAG, triglyceride concentration immediately post-diving; ΔTAG, change in triglyceride concentration between immediately post-diving and 3 hrs post-diving; AIC, Akaike Information Criterion.

**Net rate of energy gain**						
Full model	Net rate of energy gain ~ s(Experience days, k = 3, m = 2) + s(Experience days, Animal, bs = "fs", m = 2, k = 3) + Condition + s(Condition, Animal, bs = "re") + s(Animal, bs = "re")					
Final model	Net rate of energy gain ~ s(Experience days, k = 3, m = 2) + Condition + s(Condition, Animal, bs = "re") + s(Animal, bs = "re")					
	**AIC**	**ΔAIC**	**R^2**	**Family**	**n**	**Deviance explained (%)**
Final model	160.134	0.714	0.619	Gaussian	51	68.5
**$\dot{V}O_2$**						
Full model	$\dot{V}O_2$ ~ s(Experience days, k = 3, m = 2) + s(Experience days, Animal, bs = "fs", m = 2, k = 3) + s(Day of week, k = 3, m = 2) + s(Day of week, Animal, bs = "fs", m = 2, k = 3) + Condition + s(Condition, Animal, bs = "re") + s(Animal, bs = "re")					
Final model	$\dot{V}O_2$ ~ s(Experience days, k = 3, m = 2) + Condition + s(Condition, Animal, bs = "re") + s(Animal, bs = "re")					
	**AIC**	**ΔAIC**	**R^2**	**Family**	**n**	**Deviance explained (%)**
Final model	-498.674	0.463	0.605	Gaussian	53	67.3

Journal of Experimental Biology • Supplementary information

**$\dot{V}CO_2$**						
Full model	$\dot{V}CO_2$ ~ s(Experience days, k = 3, m = 2) + s(Experience days, Animal, bs = "fs", m = 2, k = 3) + s(Day of week, k = 3, m = 2) + s(Day of week, Animal, bs = "fs", m = 2, k = 3) + Condition + s(Condition, Animal, bs = "re") + s(Animal, bs = "re")					
Final model	$\dot{V}CO_2$ ~ s(Experience days, k = 3, m = 2) + Condition + s(Condition, Animal, bs = "re") + s(Animal, bs = "re")					
	**AIC**	**ΔAIC**	**R^2**	**Family**	**n**	**Deviance explained (%)**
Final model	-580.876	1.670	0.578	Gaussian (link=log)	50	64.6
**TAG**						
Full model	TAG ~ s(Experience days, k = 3, m = 2) + s(Experience days, Animal, bs = "fs", m = 2, k = 3) + s(Mass, k = 3, m = 2) + s(Mass, Animal, bs = "fs", m = 2, k = 3) + s(Time to diving sample, k = 3, m = 2) + s(Time to diving sample, Animal, bs = "fs", m = 2, k = 3) + Condition + s(Condition, Animal, bs = "re") + s(Animal, bs = "re")					
Final model	TAG ~ s(Time to diving sample, k = 3, m = 2) + s(Time to diving sample, Animal, bs = "fs", m = 2, k = 3) + Condition + s(Condition, Animal, bs = "re") + s(Animal, bs = "re")					
	**AIC**	**ΔAIC**	**R^2**	**Family**	**n**	**Deviance explained (%)**
	516.646	0.027	0.381	Gaussian (link=log)	50	52.1
**ΔTAG**						
Full model	ΔTAG ~ s(Experience days, k = 3, m = 2) + s(Experience days, Animal, bs = "fs", m = 2, k = 3) + s(Mass, k = 3, m = 2) + s(Mass, Animal, bs = "fs", m = 2, k = 3) + s(Time to diving sample, k = 3, m = 2) + s(Time to diving sample, Animal, bs = "fs", m = 2, k = 3) + s(Time to 3 hr post-diving sample, k = 3, m = 2) + s(Time to 3 hr post-diving sample, Animal, bs = "fs", m = 2, k = 3) + Condition + s(Condition, Animal, bs = "re") + s(Animal, bs = "re")					
Final model	ΔTAG ~ s(Mass, k = 3, m = 2) + s(Mass, Animal, bs = "fs", m = 2, k = 3) + Condition + s(Condition, Animal, bs = "re") + s(Animal, bs = "re")					
	**AIC**	**ΔAIC**	**R^2**	**Family**	**n**	**Deviance explained (%)**
Final model	529.354	2.001	0.326	Gaussian	49	48.4

Journal of Experimental Biology • Supplementary information

Table S3. Parametric coefficients and smooth terms of the GAMM for the response in net rate of energy gain (kJ kg^{-1}min^{-1}) to gas condition. Estimates, standard error (SE), t-values and *p*-values are presented for each parametric term. Estimated degrees of freedom (edf), reference degrees of freedom (Ref.df), F-statistics and *p*-values are presented for each smooth term. GAMM, generalised additive mixed model.

**Net rate of energy gain**				
		**Parametric coefficients**		
**Term**	**Estimate**	**SE**	**t-value**	***p*-value**
(Intercept)	5.608269	0.592937	9.458462	6.64E-12
Hypercapnic	-1.27364	0.434494	-2.93132	0.005474
Hyperoxic	-0.04494	0.406394	-0.11058	0.912483
Hypoxic	-0.80614	0.444284	-1.81447	0.076856
		**Smooth terms**		
**Term**	**edf**	**Ref.df**	**F**	***p*-value**
s(Experience days)	1.00001	1.000019	5.58707	0.022914
s(Condition, Animal)	0.000699	20	3.19E-05	0.553892
s(Animal)	4.59639	5	12.04851	<2E-16

Table S4. Parametric coefficients and smooth terms of the GAMM for the response in $\dot{V}O_2$ (L kg^{-1}min^{-1}) to gas condition. Estimates, standard error (SE), t-values and *p*-values are presented for each parametric term. Estimated degrees of freedom (edf), reference degrees of freedom (Ref.df), F-statistics and *p*-values are presented for each smooth term. GAMM, generalised additive mixed model; $\dot{V}O_2$, mass-specific rate of oxygen consumption.

**$\dot{V}O_2$**				
		**Parametric coefficients**		
**Term**	**Estimate**	**SE**	**t-value**	***p*-value**
(Intercept)	0.013542	0.000789	17.16596	7.51E-21
Hypercapnic	0.000735	0.000808	0.909258	0.368286
Hyperoxic	-0.00162	0.000759	-2.14032	0.038046
Hypoxic	0.000365	0.000815	0.447714	0.656608
		**Smooth terms**		
**Term**	**edf**	**Ref.df**	**F**	***p*-value**
s(Experience days)	1.000282	1.000558	48.29447	< 2E-16
s(Condition, Animal)	1.036462	20	0.056836	0.414532
s(Animal)	3.992981	5	4.819676	0.000658

Journal of Experimental Biology • Supplementary information

Table S5. Parametric coefficients and smooth terms of the GAMM for the response in $\dot{V}CO_2$ (L Kg^{-1} min^{-1}) to gas condition. Estimates, standard error (SE), t-values and *p*-values are presented for each parametric term. Estimated degrees of freedom (edf), reference degrees of freedom (Ref.df), F-statistics and *p*-values are presented for each smooth term. GAMM, generalised additive mixed model; $\dot{V}CO_2$, mass-specific rate of carbon dioxide production.

$\dot{V}CO_2$				
**Parametric coefficients**				
**Term**	**Estimate**	**SE**	**t-value**	**p-value**
(Intercept)	-5.44225	0.062721	-86.7688	3.48E-48
Hypercapnic	-0.19047	0.069634	-2.73528	0.009159
Hyperoxic	-0.05255	0.059708	-0.88011	0.383917
Hypoxic	0.0239	0.062094	0.3849	0.702296
**Smooth terms**				
**Term**	**edf**	**Ref.df**	**F**	**p-value**
s(Experience days)	1.000009	1.000018	36.99742	8.48E-07
s(Condition, Animal)	0.000318	20	9.35E-06	0.871687
s(Animal)	3.898736	5	3.725092	0.00158

Table S6. Model information of GLMMs for the response in circulating triglyceride concentration (mg dL^{-1}) across sampling points and lactate concentration (mmol L^{-1}) across gas conditions. The distribution family and the number of observations (*n*) are presented for each model. GLMM, generalised linear mixed model.

**Triglyceride concentration**	
**Model formula**	Triglyceride concentration ~ Sampling point + (1 | Animal)
**Family**	Gaussian (link=log)
**n**	156
**Lactate concentration**	
**Model formula**	Lactate concentration ~ Condition + (1 | Animal)
**Family**	Gaussian (link=log)
**n**	63

Journal of Experimental Biology • Supplementary information

Table S7. Fixed and random effects of the GLMM for the response in triglyceride concentration (mg dL^{-1}) to sampling point. Estimates, standard error (SE), z-values and *p*-values are presented for fixed effects. Variance and standard deviation (SD) are presented for random effects. GLMM, generalised linear mixed model.

Triglyceride concentration				
**Fixed effects**				
**Term**	**Estimate**	**SE**	**z-value**	**p-value**
(Intercept)	4.177477	0.132705	31.47935	1.7E-217
Immediately post-diving	0.503506	0.114522	4.396603	1.1E-05
3 hrs post-diving	0.831545	0.106024	7.842972	4.4E-15

Random effects		
**Term**	**Variance**	**SD**
Animal (Intercept)	0.045621	0.213591
Residual	2159.703	46.4726

Table S8. Parametric coefficients and smooth terms of the GAMM for the response in TAG (mg dL^{-1}) to gas condition. Estimates, standard error (SE), t-values and *p*-values are presented for each parametric term. Estimated degrees of freedom (edf), reference degrees of freedom (Ref.df), F-statistics and *p*-values are presented for each smooth term. GAMM, generalised additive mixed model; TAG, triglyceride concentration immediately post-diving.

TAG				
**Parametric coefficients**				
**Term**	**Estimate**	**SE**	**t-value**	**p-value**
(Intercept)	4.600995	0.140472	32.75374	2.25E-29
Hypercapnic	0.144707	0.147639	0.980138	0.333244
Hyperoxic	-0.05761	0.135228	-0.42602	0.672507
Hypoxic	0.077388	0.13257	0.583752	0.562851
**Smooth terms**				
**Term**	**edf**	**Ref.df**	**F**	**p-value**
s(Time to diving sample)	1.000036	1.000062	0.961996	0.332887
s(Time to diving sample, Animal)	7.160368	16	2.219985	0.000185
s(Condition, Animal)	0.000888	21	3.8E-05	0.522605
s(Animal)	0.00219	6	0.000334	0.082832

Table S9. Parametric coefficients and smooth terms of the GAMM for the response in ΔTAG (mg dL^{-1} to gas condition. Estimates, standard error (SE), t-values and p-values are presented for each parametric term. Estimated degrees of freedom (edf), reference degrees of freedom (Ref.df), F-statistics and *p*-values are presented for each smooth term. GAMM, generalised additive mixed model; ΔTAG, change in triglyceride concentration between immediately post-diving and 3 hrs post-diving.

**ΔTAG**				
		**Parametric coefficients**		
**Term**	**Estimate**	**SE**	**t-value**	**p-value**
(Intercept)	47.95524	15.2543	3.143719	0.003296
Hypercapnic	-13.3006	20.00649	-0.66481	0.510324
Hyperoxic	2.850582	19.59376	0.145484	0.885124
Hypoxic	-31.364	20.0903	-1.56115	0.127066
		**Smooth terms**		
**Term**	**edf**	**Ref.df**	**F**	**p-value**
s(Mass)	1.000088	1.000111	0.839312	0.365555
s(Mass, Animal)	4.368099	16	1.431982	0.001053
s(Condition, Animal)	2.914121	22	0.164316	0.262766
s(Animal)	5.96E-05	6	2.04E-06	0.928172

Table S10. Fixed and random effects of the GLMM for the response in lactate concentration (mmol L^{-1}) to gas condition. Estimates, standard error (SE), z-values and *p*-values are presented for fixed effects. Variance and standard deviation (SD) are presented for random effects. GLMM, generalised linear mixed model.

**Lactate concentration**				
		**Fixed effects**		
**Term**	**Estimate**	**SE**	**z-value**	**p-value**
(Intercept)	0.253013	0.41705	0.606673	0.544068
Ambient	1.725662	0.40919	4.217265	2.47E-05
Hypercapnic	1.382942	0.415506	3.328333	0.000874
Hyperoxic	1.640072	0.410214	3.998091	6.39E-05
Hypoxic	1.728848	0.409417	4.222705	2.41E-05

	**Random effects**	
**Term**	**Variance**	**SD**
Animal (Intercept)	0.064377	0.253726
Residual	3.50409	1.871922
